# Correction for Dietz et al., “2019 Novel Coronavirus (COVID-19) Pandemic: Built Environment Considerations To Reduce Transmission”

**DOI:** 10.1128/mSystems.00375-20

**Published:** 2020-05-05

**Authors:** Leslie Dietz, Patrick F. Horve, David A. Coil, Mark Fretz, Jonathan A. Eisen, Kevin Van Den Wymelenberg

**Affiliations:** a Biology and the Built Environment Center, University of Oregon, Eugene, Oregon, USA; b Genome Center, University of California—Davis, Davis, California, USA; c Institute for Health and the Built Environment, University of Oregon, Portland, Oregon, USA; d Department of Evolution and Ecology, University of California—Davis, Davis, California, USA; e Department of Medical Microbiology and Immunology, University of California—Davis, Davis, California, USA; f Genome Center, University of California—Davis, Davis, California, USA

## AUTHOR CORRECTION

Volume 5, no. 2, e00245-20, 2020, https://doi.org/10.1128/mSystems.00245-20. After original publication of this article, the following changes were made.

Page 5, first paragraph, line 18:

HEPA filters are rated to remove at least 99.97% of particles down to 0.3 μm (51). Most residential and commercial buildings utilize MERV-5 to MERV-11, and in critical health care settings, MERV-12 or higher and HEPA filters are used. MERV-13 filters have the potential to remove microbes and other particles ranging from 0.3 to 10.0 μm. Most viruses, including CoVs, range from 0.004 to 1.0 μm, limiting the effectiveness of these filtration techniques against pathogens such as SARS-CoV-2 (52). Furthermore, no filter system is perfect. Recently,….

was changed to

HEPA filters are rated to remove at least 99.97% of particles at 0.3 μm in size, representing the most penetrating particle size (51). Most residential and commercial buildings utilize MERV-5 to MERV-11, and in critical health care settings, MERV-13 or higher and HEPA filters are used. MERV-13 filters have the potential to remove microbes and other particles ranging from 0.3 to 10.0 μm. Most viruses, including CoVs, range from 0.004 to 1.0 μm (52). However, viruses are rarely observed as individual particles, but instead are expelled from the body already combined with water, proteins, salts, and other components as large droplets and aerosols. Thus far, SARS-CoV-2 has been observed in aerosolized particles in a spectrum of sizes including 0.25 to 0.5 μm (96), necessitating high efficiency filtration techniques to reduce the transmission potential of pathogens such as SARS-CoV-2. However,….

Page 6, third paragraph, second sentence:

Even though viral particles are too small to be contained by even the best HEPA and MERV filters, ventilation precautions can be taken to ensure the minimization of SARS-CoV-2 spread.

was changed to

Viruses are frequently found associated with larger particles (e.g., complexes with water, proteins, salts, etc.) in a range of sizes. Even though some of these particles have been identified in sizes that could potentially penetrate high efficiency filters, ventilation and filtration remain important in reducing the transmission potential of SARS-CoV-2.

Page 10, Acknowledgments, first sentence: “We thank Jason Stenson and Cassandra Moseley for comments on the manuscript” was changed to “We thank Jason Stenson, Richard Corsi, Cassandra Moseley, and Linsey Marr for comments on the manuscript.”

References: The following reference was added to the list.

96. Liu Y, Ning Z, Chen Y, Guo M, Liu Y, Gali NK, Sun L, Duan Y, Cai J, Westerdahl D, Liu X, Ho K-F, Kan H, Fu Q, Lan K. 2020. Aerodynamic characteristics and RNA concentration of SARS-CoV-2 aerosol in Wuhan hospitals during COVID-19 outbreak. bioRxiv https://doi.org/10.1101/2020.03.08.982637.

